# Pharmacological Characterization of an Antisense Knockdown Zebrafish Model of Dravet Syndrome: Inhibition of Epileptic Seizures by the Serotonin Agonist Fenfluramine

**DOI:** 10.1371/journal.pone.0125898

**Published:** 2015-05-12

**Authors:** Yifan Zhang, Angéla Kecskés, Daniëlle Copmans, Mélanie Langlois, Alexander D. Crawford, Berten Ceulemans, Lieven Lagae, Peter A. M. de Witte, Camila V. Esguerra

**Affiliations:** 1 Laboratory for Molecular Biodiscovery, Department of Pharmaceutical and Pharmacological Sciences, University of Leuven, Leuven, Belgium; 2 Luxembourg Center for Systems Biomedicine, University of Luxembourg, Esch-sur-Alzette, Luxembourg; 3 Department of Neurology-Child Neurology, University Hospital Antwerp & University of Antwerp, Antwerp, Belgium; 4 Department of Paediatric Neurology, University Hospitals Leuven, Leuven, Belgium; IGBMC/ICS, FRANCE

## Abstract

Dravet syndrome (DS) is one of the most pharmacoresistant and devastating forms of childhood epilepsy syndromes. Distinct *de novo* mutations in the *SCN1A* gene are responsible for over 80% of DS cases. While DS is largely resistant to treatment with existing anti-epileptic drugs, promising results have been obtained in clinical trials with human patients treated with the serotonin agonist fenfluramine as an add-on therapeutic. We developed a zebrafish model of DS using morpholino antisense oligomers (MOs) targeting *scn1Lab*, the zebrafish ortholog of *SCN1A*. Zebrafish larvae with an antisense knockdown of *scn1Lab* (*scn1Lab* morphants) were characterized by automated behavioral tracking and high-resolution video imaging, in addition to measuring brain activity through local field potential recordings. Our findings reveal that *scn1Lab* morphants display hyperactivity, convulsive seizure-like behavior, loss of posture, repetitive jerking and a myoclonic seizure-like pattern. The occurrence of spontaneous seizures was confirmed by local field potential recordings of the forebrain, measuring epileptiform discharges. Furthermore, we show that these larvae are remarkably sensitive to hyperthermia, similar to what has been described for mouse models of DS, as well as for human DS patients. Pharmacological evaluation revealed that sodium valproate and fenfluramine significantly reduce epileptiform discharges in *scn1Lab* morphants. Our findings for this zebrafish model of DS are in accordance with clinical data for human DS patients. To our knowledge, this is the first study demonstrating effective seizure inhibition of fenfluramine in an animal model of Dravet syndrome. Moreover, these results provide a basis for identifying novel analogs with improved activity and significantly milder or no side effects.

## Introduction

Dravet syndrome (DS) is a devastating form of childhood epilepsy that begins with prolonged seizures in the first year of life. The initial seizures are often febrile, generalized or unilateral, clonic or tonic–clonic [1]. EEG findings are mainly normal at onset, whereas epileptiform activity typically becomes evident in the second or third year or later [2]. Early development is normal, but signs of cognitive and behavioral comorbidities appear in the second year of life. Non-febrile seizures become more frequent and include convulsive status absence, myoclonic, simple and complex partial seizures [1,3,4]. Distinct *de novo* mutations in the alpha subunit of voltage-gated sodium-channel (VGSC) type 1 gene, *SCN1A*, are known to be causative of DS, mutations of which occur in 85% of Dravet patients [5]. VGSCs play an essential role in neuronal excitability by initiating and propagating the rising phase of the action potential; therefore, it is not surprising that many mutations associated with DS have been identified in *SCN1A* [6].

DS is one of the most pharmacoresistant epilepsy syndromes [7]. The main challenges are to reduce seizure frequency as much as possible, to prevent the occurrence of status epilepticus, and to optimize the development of cognitive functions [7]. Stiripentol is the only compound that has shown some efficacy in DS patients through two independent randomized placebo-controlled add-on trials, when combined with valproate and clobazam [8,9]. However, these agents do not yield complete seizure freedom and may cause adverse side effects [10]. New, effective antiepileptic drugs (AEDs) with possibly novel mechanisms of action would therefore significantly improve current treatment options [11,12]. In a recent clinical study, the anticonvulsant activity of fenfluramine was tested. Seventy percent of children treated with add-on fenfluramine were seizure-free for more than 1 year, which is better than any other treatment option tried for Dravet syndrome to date [13]. Therefore, fenfluramine is proposed as a new and potent anti-epileptic add-on drug in DS. Fenfluramine is a potent 5-HT (serotonin) releaser. Serotonin is able to activate multiple 5-HT receptor subtypes, of which 14 different ones have been described in humans. Moreover, the N-dealkylated metabolite of fenfluramine, i.e. norfenfluramine, displays high affinity and activity at the 5-HT2B and 5-HT2C receptor subtypes [14]. Which 5-HT receptor subtypes are involved in the anti-epileptic effect of fenfluramine is presently unknown. 5-HT2C receptor agonists trigger appetite suppression [15]. Conversely, the activation of 5-HT2B receptors is associated with cardiac valve injury [16]. Thus, the current challenge is to determine whether the mechanism underlying fenfluramine’s anticonvulsant activity is the same or different from the mechanism leading to valvulopathies. It should be noted, however, that the dose used for treating DS children in clinical studies was considerably lower than the fenfluramine dose, used in the treatment of obesity that was associated with cardiac valve injury. To date, however, fenfluramine has not been evaluated in animal models of DS.

Several mouse DS models, and more recently also a zebrafish DS model, have been described that strikingly recapitulate the DS phenotype including age and temperature dependence of spontaneous epileptic seizures and ataxia [17–21], proving the possibility to study the syndrome in animal models and to potentially use these models to screen for novel therapeutics. Due to a partial genome duplication in teleost fishes, zebrafish express two paralogs of each member of the *VGSC* gene family: *scn1Laa* and *scn1Lab*, *scn5Laa* and *scn5Lab*, and *scn8aa* and *scn8ab*, which encode Na_v_1.1La and Na_v_1.1Lb, Na_v_1.5La and Na_v_1.5Lb, Na_v_1.6a and Na_v_1.6b, respectively [22]. Previous phylogenetic and expression pattern analyses have indicated that zebrafish *scn1Lab* is evolutionarily most closely related to the mammalian *SCN1A*, *SCN2A*, and *SCN3A* genes [23]. It was shown that *scn1Lab* displays neuronal expression pattern in both zebrafish embryos (24 hours post-fertilization) and adults [22,23]. The zebrafish *scn1Lab* mutant, *double indemnity (didy)*, was discovered in an ENU-mutagenesis screen and has been investigated in two studies. Schoonheim *et al*. characterized the *didy* phenotype based on a defect in sustaining saccades during the optokinetic response [24], whereas Baraban *et al*. recently demonstrated that *scn1Lab* mutants exhibit spontaneous seizures [21]. These *didy* mutants were used to identify compounds that rescue the phenotype, such as clemizole, an FDA-approved drug, that effectively inhibits spontaneous convulsive seizures both at behavioral and electrographic level [21].

In order to be able to rapidly analyze the potential anti-epileptic activity of fenfluramine, we developed a zebrafish model for this disorder using morpholino antisense oligomers targeting *scn1Lab*, the zebrafish ortholog of *SCN1A*. Here we describe the pathophysiological and pharmacological characterization of this zebrafish DS model, which displays abnormal locomotor behavior, recurrent electrographic discharges and age-dependent hyperthermia sensitivity. Seizure-like locomotor behavior and electrographic discharges in this model are effectively treated with fenfluramine, thereby providing further evidence for this drug candidate as a potential therapeutic for DS and demonstrating the utility of this zebrafish DS model for rapidly evaluating potential new small-molecule therapeutics for DS.

## Methods

### Zebrafish maintenance and breeding

Adult zebrafish (*Danio rerio*) stocks of the AB strain (Zebrafish International Resource Center, Oregon, USA) were maintained at 28.0°C, on a 14/10 hour light/dark cycle under standard aquaculture conditions. Fertilized eggs were collected via natural spawning. Embryos and larvae were kept on a 14/10 hour light/dark cycle in embryo medium: 1.5 mM HEPES, pH 7.6, 17.4 mM NaCl, 0.21 mM KCl, 0.12 mM MgSO_4_, and 0.18 mM Ca(NO_3_)_2_ in an incubator at 28.0°C. All zebrafish experiments carried out were approved by the Ethics Committee of the University of Leuven (Ethische Commissie van de KU Leuven, approval number (061/2013) and by the Belgian Federal Department of Public Health, Food Safety & Environment (Federale Overheidsdienst Volksgezondheid, Veiligheid van de Voedselketen en Leefmileu, approval number LA1210199).

### Antisense morpholino oligomers (MO) and microinjections

9 ng of a translation blocking MO (ATG MO: 5’-CTGAGCAGCCATATTGACATCCTGC-3’) was used to achieve partial knockdown of zebrafish *scn1Lab*. Standard control MO (5'-CCTCTTACCTCAGTTACAATTTATA) or randomized 25-N MO was used as a negative control (CTRL MO) (9 ng). All MOs were designed and synthesized by GeneTools LLC (Philomath, Oregon, USA) and injected into one- to two-cell stage embryos.

### Larval locomotor behavior

In order to assess the locomotor behavior of Dravet syndrome morphant model, *scn1Lab* morphants and control larvae were placed in a 96-well plate in 200 μL of embryo medium from 3 to 7 days post fertilization (dpf). Each day the larvae were tracked in an automated tracking device (ZebraBox apparatus; Viewpoint, Lyon, France) for one hour (30-minute integration interval), followed by a 30-minute chamber of habituation under white light, as all recordings were performed at the same time during daytime period. The total movement was recorded, then, quantified using ZebraLab software (Viewpoint, Lyon, France) and plotted in “actinteg” units, which is the sum of all pixel changes detected during the experimental period [25]. Data were pooled together from three independent experiments with twelve larvae per injection condition.

### Generation of zebrafish larvae with non-inflated swim bladder

Dechorionated 2-dpf control larvae were placed in a 6-well plate filled to the brim with embryo medium and covered with a glass lid in order to prevent larvae from being exposed to air, thereby preventing them inflating their swim bladders. At 5 dpf, larvae were tracked in an automated tracking device as described, using similar conditions that prevented them to fill their swim bladders with air.

### Local field potential recordings

Open-field recordings were obtained from zebrafish larval forebrain at 5 dpf at 24°C [26]. A glass electrode, connected to a high-impedance amplifier, was filled with artificial cerebrospinal fluid (124 mM NaCl, 2 mM KCl, 2 mM MgSO_4_, 2 mM CaCl_2_, 1.25 mM KH_2_PO_4_, 26 mM NaHCO_3_ and 10 mM glucose). A larva was then embedded in 2% low-melting-point agarose (Invitrogen) and the glass electrode (2–7 MΩ) placed into the forebrain of the larva. The recordings were performed in current clamp mode with parameters: low-pass filtered at 1 kHz, high-pass filtered 0.1 Hz, digital gain 10 and sampling interval 10 μs (MultiClamp 700B amplifier, Digidata 1440A digitizer, both Axon instruments, USA). Single recordings were performed for ten minutes. Both fenfluramine and sodium valproate were applied 24 hours prior to extracellular recordings. In order to inhibit the spontaneous epileptiform activity of *scn1Lab* morphants, they were incubated with a mixture of 20 μM 6-cyano-7-nitroquinoxaline-2,3-dione (CNQX), and 50 μM DL-2-amino-5-phosphonopentanoic acid (APV), 30 min prior to forebrain LFP recordings. Control larvae were exposed to 20 mM pentylenetetrazole (PTZ) to induce epileptiform activity or to a mixture containing 20 mM PTZ, 20 μM CNQX and 50 μM APV, 30 min prior to midbrain LFP recordings. Spontaneous epileptiform events were taken into account when the amplitude exceeded three times the background noise. The analysis of spikes was carried out using Clampfit 10.2 software (Molecular Devices Corporation, USA).

### Hyperthermia-induced abnormalities

*scn1Lab* morphants and control larvae (8 larvae per condition between 3 and 7 dpf) were placed in 0.2 ml PCR tubes filled with 50 μL embryo medium, exposed to a rapid (ca. 10 s) temperature change from 28°C to 39°C and maintained at 39°C for 10 min in a thermal cycler. Afterwards, larvae were transferred to a 6-well plate at 28°C on a 14/10 hour light/dark cycle under standard aquaculture conditions and observed for 24 hours. In order to evaluate the effect of fenfluramine and sodium valproate on the outcome, 4-dpf *scn1Lab* morphants and control larvae were exposed to the compounds 24 hours prior to heatshock treatment. To quantify the severity of hyperthermia-induced abnormalities, 24 hours after heatshock, cumulative scoring was used. Normal behavior was scored as 0, decreased touch response and partial loss of posture as 1, absent touch response and complete loss of posture as 2, and death as 3. To obtain a cumulative score, the number of larvae was multiplied by the value that corresponded to the level of severity. Data for hyperthermia-induced abnormalities were pooled together from three independent experiments.

### Drugs

Carbamazepine (CBZ), topiramate (TPR), stiripentol (STP), pentylenetetrazole (PTZ), 6-cyano-7-nitroquinoxaline-2,3-dione (CNQX) and DL-2-amino-5-phosphonopentanoic acid (APV) were purchased from Sigma-Aldrich, sodium valproate (VPA) from Sanofi-Aventis, clobazam (CLB) from Lipomed AG, Switzerland and fenfluramine (FA) from Peak International Products B.V. Compounds were dissolved in DMSO and diluted in embryo medium to achieve a final DMSO concentration of 1% w/v, which served as a vehicle control (VHC). In case of treatment for the hyperthermia-experiment, a final DMSO concentration of 0.1% w/v was used.

### Determination of maximal tolerated concentration (MTC)

Freely swimming 3 dpf *scn1Lab* morphants and control larvae were incubated in 96-well plate format with AEDs or VHC at 28°C on a 14/10 hour light/dark cycle under standard aquaculture conditions (medium was replenished after 24 hours of incubation at 4 dpf). Each larva was individually checked under the microscope for the following signs of acute locomotor impairment and toxicity, such as decreased or absent touch response, loss of posture, body deformation, slow or absent heartbeat, and death after 24 hours (4 dpf) and 48 hours (5 dpf) of incubation. A larva was considered normal if it could cover a distance twice its body length. A shorter distance traveled or movement in the same place was scored as a decreased or impaired touch response. No visible movement upon a touch stimulus was counted as absent response. Thus, the MTC was defined as the maximum concentration, which did not cause death, dysmorphology, abnormal heart rate and where not more than two out of twelve larvae exhibited any sign of locomotor impairment including absent touch response and loss of balance after 24 and 48 hours of incubation. All vehicle-treated (VHC), control larvae displayed no signs of locomotor impairment or toxicity and had normal heart rates after 24 and 48 hours of incubation.

### Pharmacological evaluation of larval locomotor behavior

Freely swimming 3 dpf *scn1Lab* morphants and control larvae were pre-incubated in 200 μl of different concentrations of AEDs (at or below the MTC) or VHC for 24 hours in individual wells of a 96-well plate at 28°C on a 14/10 hour light/dark cycle under standard aquaculture conditions. After 24 hours of incubation and 30-minute chamber habituation 4 dpf larvae were tracked for locomotor behavior for one hour (30 minutes integration interval) under white light. After tracking, the embryo medium with AEDs was replenished for another 24 hours (with same concentrations). Same tracking experiments were performed with 5 dpf larvae after 48 hours of incubation. The total locomotor activity was quantified using ZebraLab software (Viewpoint, Lyon, France) and plotted in “actinteg” units. Data were pooled together from two (CBZ, CLB, STP, TOP) or three (VPA, FA) independent experiments with twelve larvae per injection condition.

### Video recordings

Individual *scn1Lab* morphant or control larva was placed into a glass well (inner diameter: 7 mm, depth: 2 mm) filled with embryo medium and filmed for 20 seconds using a Carl Zeiss Stemi 2000-C stereomicroscope equipped with digital camera (InSight 2Mp, Diagnostic Instruments) run by VisiView software (ID 1216) (Exposure time: 10 msec, time interval: 200 msec). Each larva was kept at 28°C by the following method: the glass well with the larva was warmed up by a home-made glass-heating element (connected to a water bath) in which water was circulated by a peristaltic pump. Temperature of the embryo medium in the glass well was measured at each recording with a micro-thermometer probe (Testo AG, Germany).

### Imaging

*scn1Lab* morphants and control larvae were photographed using a Leica MZ10 F stereomicroscope equipped with a DFC310 FX digital camera run by Leica Application Suite software (version 3.6.0).

### Statistical analysis

Statistical analyses were performed using Mann–Whitney test, (larval locomotor behavior), Student’s two-tailed unpaired *t*-test or Mann–Whitney–Rank sum test for data that failed the normality test, as appropriate (local field potential recordings). Outliers of the assessment of data for pharmacological evaluation of larval locomotor behavior were removed using Iterative Grubbs’ method. Data were then analyzed by one-way ANOVA followed by Dunnett’s multiple comparison test with GraphPad Prism 6.01 software.

## Results and Discussion

### Characterization of the zebrafish *scn1Lab* morphant phenotype

#### Antisense morpholino knockdown

A Dravet syndrome morphant model was generated by knocking down the zebrafish *scn1Lab* gene using morpholino antisense oligomer. The MO targeted the region spanning the 5’UTR and translational start site of *scn1Lab* mRNA (ATG MO). Ideally, the level of knock down via translation inhibition with ATG MO should be evaluated by Western blotting. However, no proper antibody exists currently to determine the antisense effects of ATG MO [21]. Two different antibodies were tested. Unfortunately, none of them showed specificity against zebrafish Na_v_1.1Lb (Abcam—Anti-Scn1a antibody (ab24820) and Biorbyt Anti-SCN1A antibody (orb13681), data not shown).

#### Morphological description

**Fig 1:**
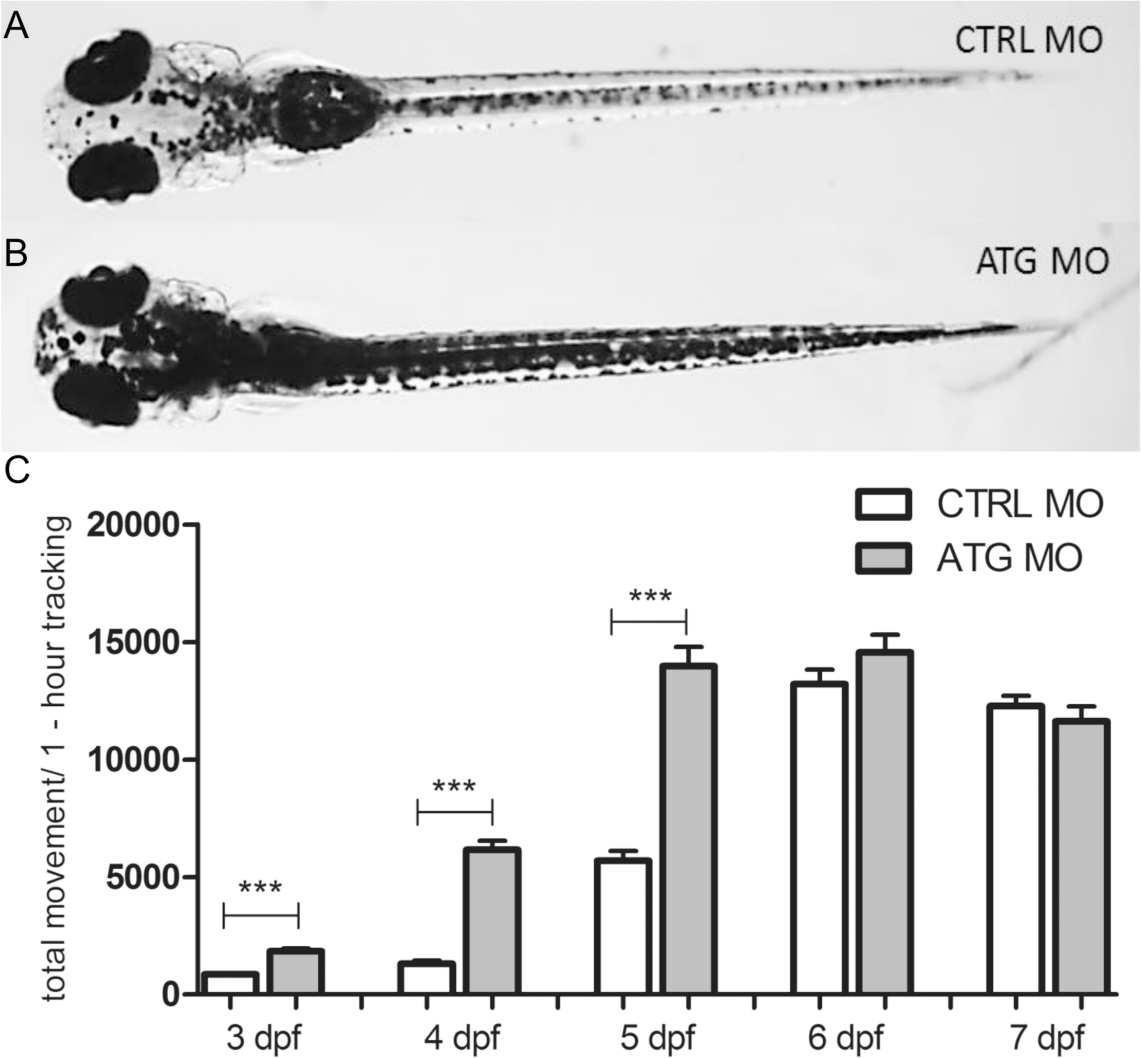
Morphology and larval locomotor activity of *scn1Lab* ATG morphants. (A) Dorsal view of a representative 5 dpf CTRL MO-injected larva. (B) Dorsal view of a representative 5 dpf ATG MO-injected larva. (C) The y-axis depicts the total larval locomotor activity of ATG MO- compared to CTRL MO-injected larvae over a one-hour tracking experiment (30-minute integration time). Data were pooled from three independent experiments with 12 larvae per injection condition. Statistical analysis was performed using Mann-Whitney test. Error bars on all graphs represent the standard error of mean (SEM). *** P ≤ 0.001.

We performed visual inspection of *scn1Lab* morphants from 1 dpf to 7 dpf, and observed that between 3 and 7 dpf, larvae failed to inflate their swim bladder, were hyperpigmented and displayed slightly curved body axes (data shown at 5 dpf, Fig 1B). Moreover, no signs of necrosis, axis truncation, pericardial edemas or any other dysmorphologies were observed. This observed phenotype is identical to the already described findings for the *scn1Lab* mutant [21,24]. Thus, *scn1Lab* morphants phenocopied the reported genetic *scn1Lab* mutant, underscoring the specificity of the MO. At all stages studied, control embryos and larvae displayed normal phenotypes (data shown at 5 dpf, Fig 1A).

#### Assessment of larval locomotor behavior

**Fig 2:**
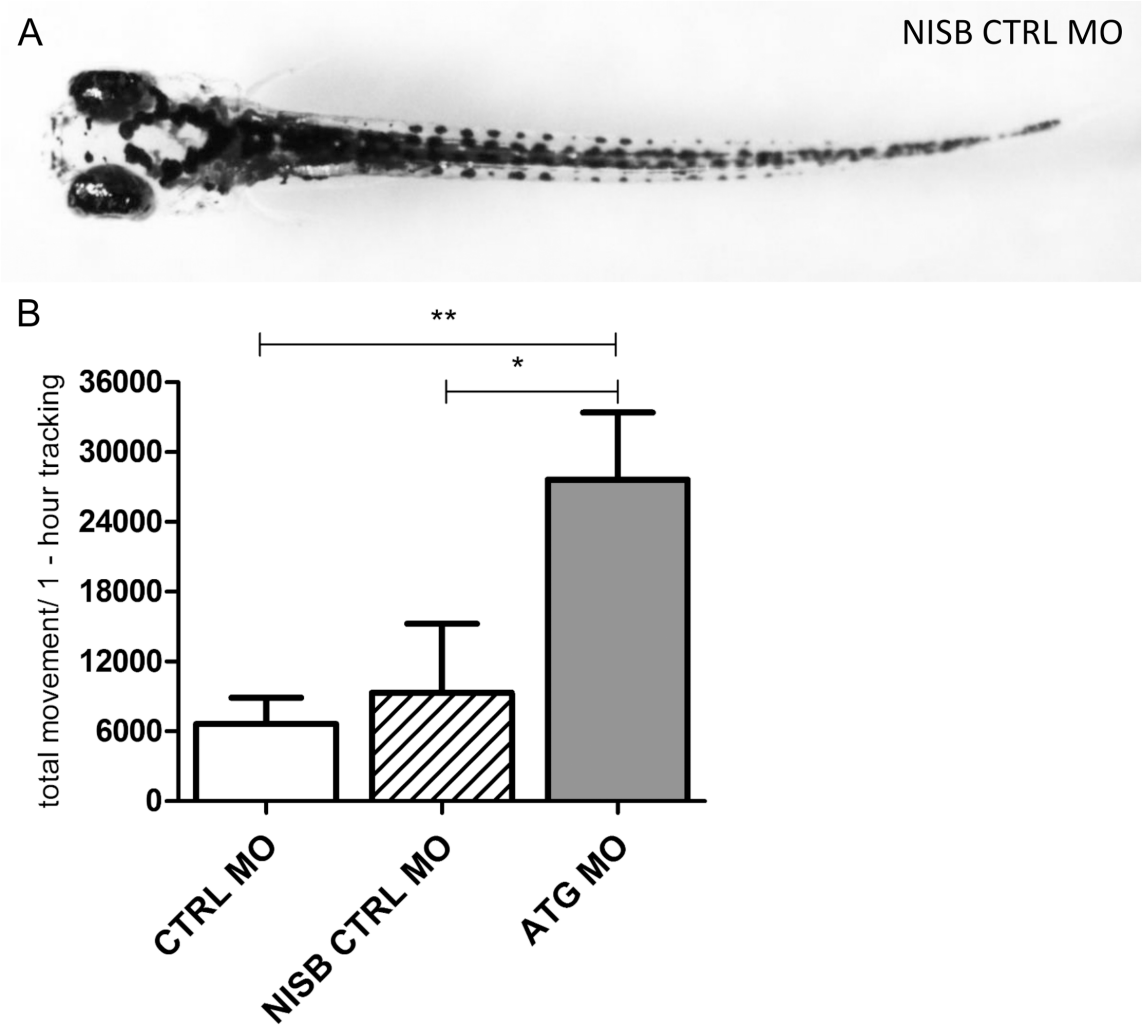
Morphology and larval locomotor activity of *scn1Lab* ATG morphants in comparison to control larvae with non-inflated swim bladder. (A) Dorsal view of a representative 5-dpf CTRL MO-injected larva with non-inflated swim bladder (NISB CTRL MO). (B) The y-axis depicts the total larval locomotor activity of ATG MO- compared to CTRL MO-injected larvae and CTRL MO-injected larvae win non-inflated swim bladder (NISB CTRL MO) over a one-hour tracking experiment (30-minute integration time). Data were analyzed from one experiment with 8–12 larvae per injection condition. Statistical analysis was performed using Dunnett's multiple comparisons test. Error bars on all graphs represent standard error of mean (SEM). * P ≤ 0.05, ** P ≤ 0.01.

An automated video-based behavioral tracking system (ZebraBox, ViewPoint, France) was used to simultaneously monitor and quantify the locomotor activity of freely swimming *scn1Lab* morphant and control larvae individually arrayed in 96-well plate from 3 dpf to 7 dpf. The *scn1Lab* morphants displayed spontaneously increased total movement as compared to control larvae. The increase of total movement was initially observed at 3 dpf and became more pronounced at 4 and 5 dpf (p<0.001) (Fig 1C), thus showing Stage I seizure-like behavior as described previously [27]. In order to characterize the locomotor behavior of the *scn1Lab* morphant in more detail, we performed higher-resolution video recording to capture more subtle larval seizure behaviors not detected by the automated tracker. *scn1Lab* morphant larvae displayed not only increased total movement but also abnormal behavior. Larvae displayed jerking behavior and sudden stiffening and relaxation of the entire body (S1 and S2 Videos, shown at 5 and 6 dpf). Other phenotypic traits that typified the *scn1Lab* morphant larvae were a non-inflated swim bladder and a bent axis (Fig 1B), as observed before in the *scn1Lab*/didy mutant [24],

In order to investigate whether the balance defects and/or abnormal behavior were caused by the swim bladder defect, we generated control larvae (CTRL MO-injected larvae) with non-inflated swim bladders (NISB CTRL MO). Under normal circumstances, 2-3-dpf larvae show swim-up behavior to inflate the swim bladder at the surface, a spontaneous behavior which occurs almost immediately after hatching [28]. By preventing the larvae to surface, we were able to prevent larvae to inflate their swim bladders. Interestingly, we observed up to 6 dpf that NISB-control larvae also showed a slightly bent axis (data shown at 5 dpf, Fig 2A) and altered behavior such as difficulties in maintaining balance (transient loss of posture) and subtle movements, similar to *scn1Lab* morphants (S3 and S4 Videos, shown at 5 and 6 dpf). These locomotor defects were never observed in any of the control larvae (with inflated swim bladder) (S5 and S6 Videos, shown at 5 and 6 dpf).

Then, at 5 dpf the total movement of NISB-control larvae was compared to control siblings with normally inflated swim bladders and *scn1Lab* morphant larvae, which have morpholino-induced non-inflated swim bladders. Importantly, we found that the total movement of control larvae was comparable to NISB-control larvae (p = 0.9333, Fig 2B). Hence, we concluded that the abnormal hyperactive behavior of the *scn1Lab* morphants between 3 and 5 dpf is not due to swim bladder deficiency, but very likely due to abnormal brain activity as confirmed by local field potential (LFP) measurements (see further). Conversely, the abnormal behavior seen in 6 dpf *scn1Lab* morphants (shown in S2 Video), as also observed in the control larvae with non-inflated swim bladders, can be related to the lack of the swim bladders (shown in S3 and S4 Videos). The latter movements, at least in part due to the lack of swim bladders, are not picked up by the automated tracker (Fig 1C: non-significant difference at 6 and 7 dpf), whereas the hyperactivity of the *scn1Lab* morphants (i.e. not related to the lack of the swim bladders), can easily be observed between 3 and 5 dpf (Fig 1C and S1 Video).

#### Local field potential recordings

**Fig 3:**
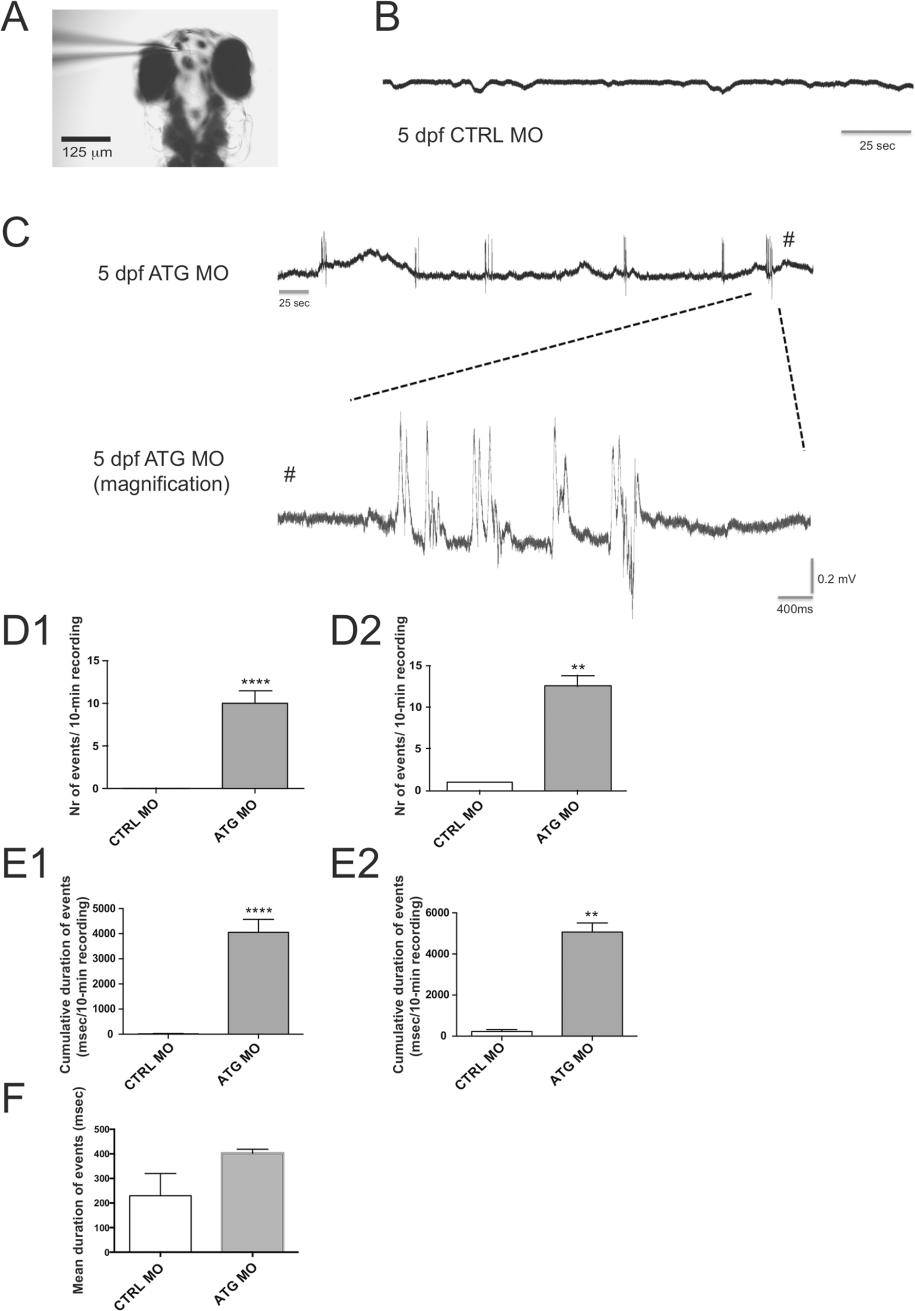
Spontaneous electrographic activity recorded from 5 dpf ATG MO- and CTRL MO-injected larvae. (A) Representative recording configuration of an agar-immobilized zebrafish at 5 dpf. Note the recording electrode placed in the forebrain. (B) Extracellular recordings from the forebrain of 5 dpf CTRL-MO larvae. (C) Representative epileptiform activities of 5 dpf ATG MO-injected larvae displaying polyspiking discharges. Top trace represents a typical epileptiform pattern as seen in gap-free recordings. Bottom trace shows high-resolution magnification of epileptiform events mentioned above. (D) Occurrence of epileptiform events in CTRL MO- and ATG MO-injected larvae. (D1) All larvae: CTRL MO 0.07±0.05 *vs* ATG MO 10.03±1.38 events/recording (n = 28 and 30 larvae, respectively). (D2) Seizure-positive larvae: CTRL MO 1.0±0.0 *vs* ATG MO 12.54±1.27 events/recording (n = 2 and 24 larvae, 2 and 302 events analyzed, respectively). (E) Cumulative duration of epileptiform events in CTRL MO- and ATG MO-injected larvae. (E1) All larvae: CTRL MO 16.4±12.3 *vs* ATG MO 4053±515 msec/10 min-recording (n = 28 and 30 larvae, respectively). (E2) Seizure-positive larvae: CTRL MO 290±91 *vs* ATG MO 5066±441 events/recording (n = 2 and 24 larvae, respectively). (F) Mean duration of electrographic activity recorded from 5 dpf CTRL MO- and ATG MO-injected larvae. ATG MO, 402.6±16.4 *vs* CTRL MO 230±91 msec (n = 302 and 2 events from 30 and 2 larvae, respectively, *P* = 0.3929). Statistical analysis was performed using Student’s unpaired *t*-test or Mann–Whitney test for data that failed the normality test, as appropriate. Error bars on all graphs represent standard error of mean (SEM). ** P ≤ 0.01, **** P≤0.0001.

To confirm that *scn1Lab* knockdown resulted in abnormal brain activity, we performed LFP recordings in larval forebrain on 5 dpf *scn1Lab* morphants and control larvae (Fig 3A). Epileptiform paroxysmal events consisted of polyspiking discharges with amplitudes equal to or exceeding threefold baseline (Fig 3C). Such recurrent spontaneous epileptiform events occurred in 24/30 (80%) of *scn1Lab* morphants. Controls displayed baseline activity (Fig 3B and 3D1), whereas 2 out of 28 control larvae showed a single epileptiform-like event (Fig 3C and 3D1). In seizure-positive larvae, the occurrence of polyspiking discharges was significantly higher in *scn1Lab* morphants, with a mean frequency of 12.5 events/10 min recording, in comparison to control larvae who only displayed one unique epileptiform event (Fig 3D2). As a consequence, the cumulative duration of epileptiform events, i.e. the fraction of time spent in epileptic activity was significantly higher in *scn1Lab* morphants compared to control larvae (all larvae: Fig 3E1, seizure positive larvae: E2), whereas the mean duration of epileptiform events was not affected (Fig 3F).

**Fig 4:**
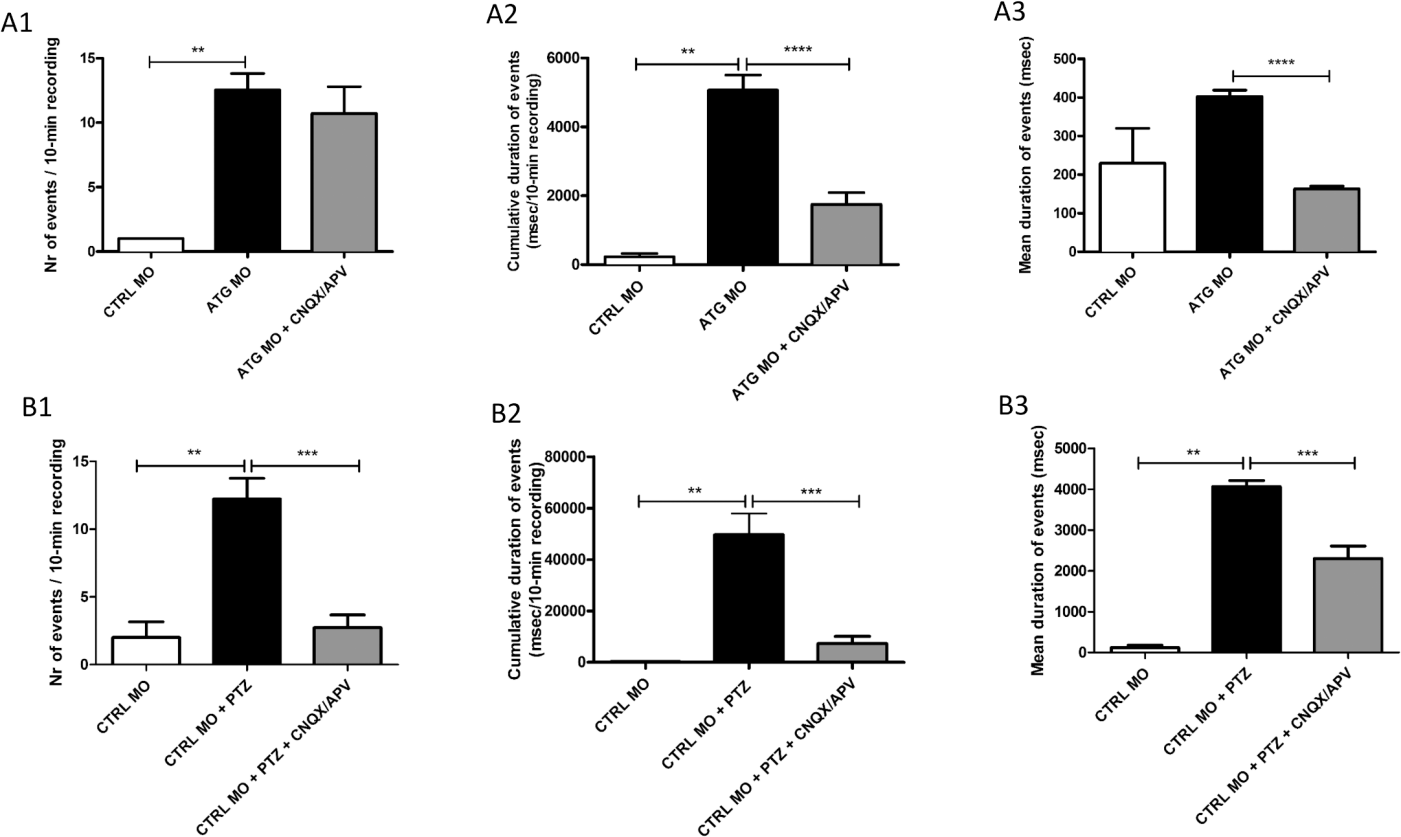
Inhibition of spontaneous or PTZ-induced electrographic discharges recorded from 5-dpf ATG MO- or CTRL MO-injected larvae after application of CNQX/APV. (A1) Occurrence of polyspiking discharges recorded in ATG MO-injected larvae after 30 minutes treatment with 20 μM CNQX/50 μM APV in all larvae (CTRL MO, 1.0 ± 0 *vs* ATG MO, 12.5 ± 6.2 *vs* ATG MO+CNQX/APV 10.7 ± 5.5 events/10-min recording, n = 3, 24 and 7 larvae respectively). (A2) Cumulative durations of polyspiking discharges recorded in ATG MO-injected larvae after 30 minutes treatment with 20 μM CNQX/50 μM APV in all larvae (CTRL MO, 229.7 ±128.1 *vs* ATG MO, 5066 ± 2162 *vs* ATG MO+CNQX/APV 1749 ± 904.2 msec/10-min recording). (A3) Mean durations of polyspiking discharges recorded in ATG MO-injected larvae after 30 minutes treatment with 20 μM CNQX/50 μM APV in all larvae (CTRL MO, 229.7±128.1 *vs* ATG MO, 402.6 ± 285.3 *vs* ATG MO+CNQX/APV 163.3 ± 60.8 msec, n = 2, 302 and 75 events, respectively). (B1) Occurrence of polyspiking discharges recorded in CTRL MO-injected larvae after 30 minutes treatment with 20 mM PTZ and 20 μM CNQX/50 μM APV with 20 mM PTZ in all larvae (CTRL MO, 2.0 ± 2.0 *vs* CTRL MO+PTZ, 12.2 ± 4.6 *vs* CTRL MO+PTZ+CNQX/APV 2.7 ± 3.1 events/10-min recording, n = 3, 9 and 11 larvae respectively). (B2) Cumulative durations of polyspiking discharges recorded in CTRL MO-injected larvae after 30 minutes treatment with 20 mM PTZ and 20 μM CNQX/50 μM APV with 20 mM PTZ in all larvae (CTRL MO, 320.3 ± 279.7 *vs* CTRL MO+PTZ, 49640 ± 24833 *vs* CTRL MO+PTZ+CNQX/APV 7321 ± 9257 msec/10-min recording). (B3) Mean duration of polyspiking discharges recorded in CTRL MO-injected larvae after 30 minutes treatment with 20 mM PTZ and 20 μM CNQX/50 μM APV with 20 mM PTZ in all larvae (CTRL MO, 117.2 ± 111.7 *vs* CTRL MO+PTZ, 4061 ± 1548 *vs* CTRL MO+PTZ+CNQX/APV 2301 ± 1810 msec, n = 3, 110 and 35 events, respectively). Statistical analysis was performed using Student’s unpaired *t*-test or Mann–Whitney test for data that failed the normality test, as appropriate. Error bars on all graphs represent standard error of mean (SEM). ** P ≤ 0.01, **** P≤0.0001.

To confirm that the recorded brain activity are indeed synchronized neuronal discharges, blockers of glutamate-mediated synaptic transmission were applied to *scn1Lab* morphants. *scn1Lab* morphants were incubated with a mixture of 20 μM 6-cyano-7-nitroquinoxaline-2,3-dione (CNQX), an AMPA/kainate receptor antagonist, and 50 μM DL-2-amino-5-phosphonopentanoic acid (APV), an NMDA receptor antagonist, 30 min prior to forebrain LFP recordings [27]. Although application of CNQX/APV did not affect the occurrence of polyspiking discharges (Fig 4A1), cumulative duration of events, Fig 4A2) and mean duration of events Fig 4A3) were significantly reduced. These results show that recorded polyspiking discharges are strongly inhibited by glutamate receptor blockers and thus dependent on synaptic activities. Therefore, it was concluded that recorded abnormal brain activity from *scn1Lab* morphants is epileptiform.

As a positive control, seizures were induced in control larvae by addition of 20 mM pentylenetetrazole (PTZ), a commonly used chemoconvulsant, 30 min prior to midbrain LFP recordings [27]. Again the inhibitory effect of CNQX/APV application was tested. Blockers were applied together with PTZ to control larvae, 30 min prior to midbrain LFP recordings. Application of CNQX/APV significantly reduced occurrence of polyspiking discharges (Fig 4B1), cumulative duration of events Fig 4B2) and mean duration of events (Fig 4B3).

#### Hyperthermia-induced abnormalities

Dravet syndrome frequently starts with convulsive seizures during the first year of life, which is triggered by elevated body temperature (due for instance, to vaccination, infection or a hot bath) [1,29,30]. Oakley *et al*. also observed temperature-sensitive seizures in a mouse model of Dravet syndrome [20]. In line with these findings, we investigated whether hyperthermia *per se* could influence larval behavior and cause abnormalities and/or death in *scn1Lab* morphants by generating a rapid temperature shift in the embryo medium from 28°C to 39°C, which was maintained for ten minutes. At lower temperatures, heatshock-related abnormalities were not observed (data not shown).

**Fig 5:**
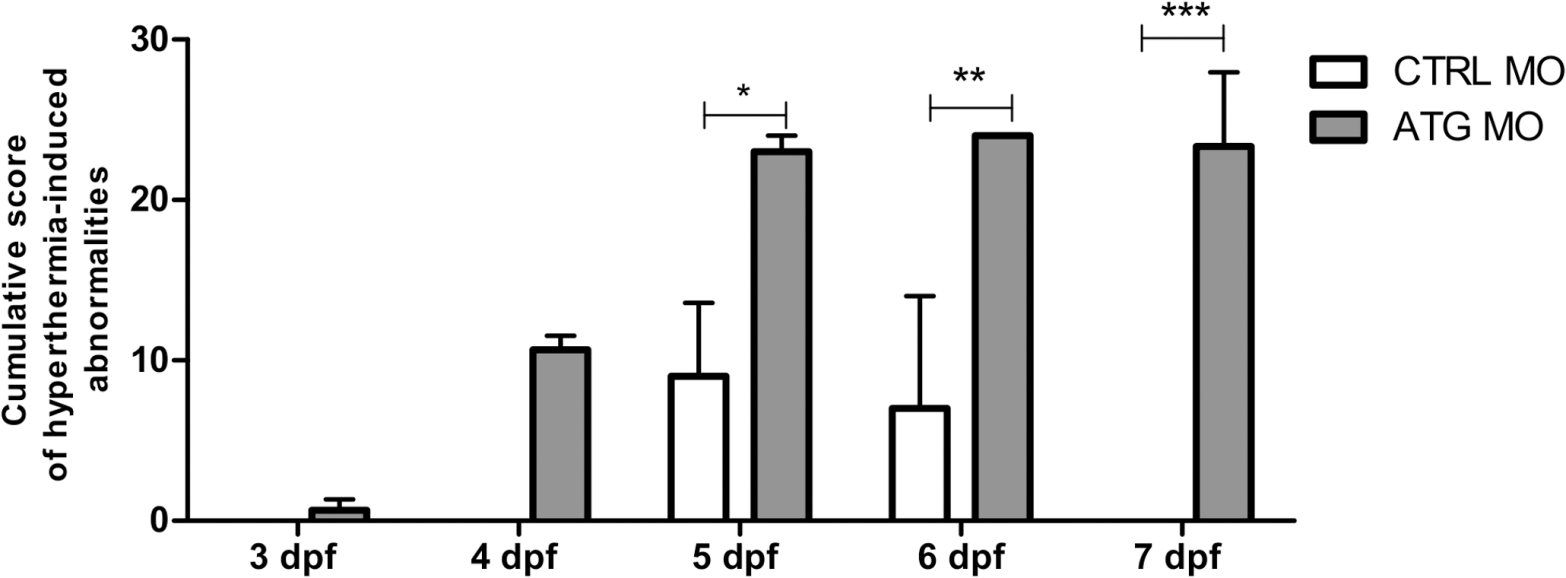
Confirmation of hyperthermia-induced abnormalities. The y-axis depicts the cumulative score to quantify the severity of hyperthermia-induced abnormalities 24 hours after heatshock. Normal behavior was scored as 0, decreased touch response and partial loss of posture as 1, absent touch response and complete loss of posture as 2, and death as 3. The number of larvae was multiplied by the value corresponding to the level of severity. The x-axis corresponds to CTRL MO- and ATG MO-injected larvae from 3 dpf to 7 dpf (day of heatshock). Data of graphs are pooled together from three independent experiments with 8 larvae per condition. Statistical analysis was performed using two-way ANOVA with Bonferroni post-tests. Error bars on all graphs represent standard error of mean (SEM). * P ≤ 0.05, ** P ≤ 0.01, *** P≤0.001.

To quantify the severity of hyperthermia-induced abnormalities, cumulative scoring was used. Normal behavior was scored as 0, decreased touch response and partial loss of posture as 1, absent touch response and complete loss of posture as 2, and death as 3. To obtain a cumulative score, the number of larvae was multiplied by the value that corresponded to the level of severity, thus, the higher the value plotted, the more severe the observed phenotype was per condition. We observed that *scn1Lab* morphants were extremely sensitive to heat shock compared to control larvae between 5 and 7 dpf (Fig 5).

Seizures of DS typically do not begin before 5 months of age suggesting a strong correlation between time of onset and brain development [1,20]. Moreover, it is also known that febrile seizures are difficult to induce in rat pups due to the immaturity of the brain and immune system [31,32]. Interestingly, we could detect a clear age-dependent transition for hyperthermia-induced death in *scn1Lab* morphants as was also shown in a mouse model of Dravet syndrome [20]. To be more specific, we could not detect a significant difference in hyperthermia-induced abnormalities between *scn1Lab* morphants and control larvae before 5 dpf, while this was apparent between 5 and 7 dpf.

### Pharmacological evaluation of zebrafish Dravet morphant model

Commercially available AEDs known to modulate seizure progression of Dravet syndrome patients [33,34] were chosen to carry out a pharmacological evaluation of *scn1Lab* morphants. We tested carbamazepine (CBZ), clobazam (CLB), stiripentol (STP), topiramate (TOP), sodium valproate (VPA) and fenfluramine (FA) to evaluate their ability to rescue or worsen the abnormal larval hyperactivity of *scn1Lab* morphants to determine whether the tested AEDs would ameliorate or worsen the Dravet morphant phenotype.

#### Assessment of larval locomotor response to AEDs

**Fig 6:**
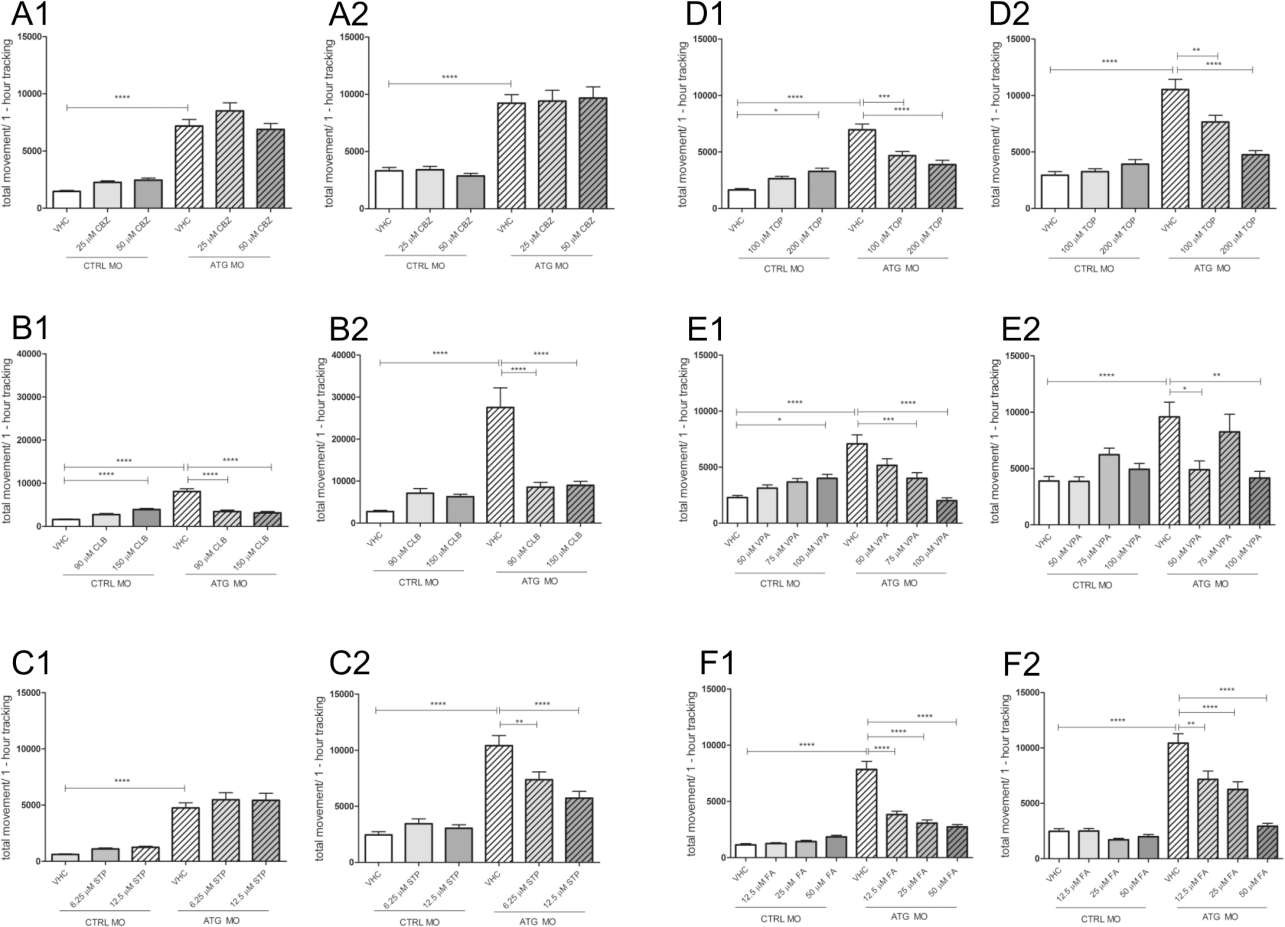
Effect of carbamazepine (CBZ), clobazam (CLB), stiripentol (STP), topiramate (TOP), sodium valproate (VPA) and fenfluramine (FA) on total larval locomotor movement of 4 and 5 dpf CTRL MO- and ATG MO-injected larvae. (A1, B1, C1, D1, E1, F1) 4 dpf CTRL MO- and ATG MO-injected larvae after 24 hours of incubation of vehicle (VHC) and CBZ, CLB, STP, TOP, VPA or FA, respectively. (A2, B2, C2, D2, E2, F2) 5 dpf CTRL MO- and ATG MO-injected larvae after 48 hours of incubation of vehicle (VHC) and CBZ, CLB, STP, TOP, VPA or FA, respectively. Total movement is expressed in “actinteg” units over 1 hour of tracking experiment (30-min intervals). Data were pooled from three independent experiments with twelve larvae per injection condition. Statistical analysis was performed using one-way analysis of variance (ANOVA) followed by Dunnett’s test for multiple comparisons. Error bars on all graphs represent standard error of mean (SEM). * P ≤ 0.05, ** P ≤ 0.01, *** P≤0.001, **** P≤0.0001.

The locomotor behavior of *scn1Lab* morphants in response to different AEDs was investigated using the ZebraBox system between 4 and 5 dpf, since they displayed significantly higher total movement compared to control larvae (Fig 1C). Therefore, all the pharmacological experiments were carried out during this developmental period to observe the highest difference. With the replenishment of AEDs after 24 hours we wanted to investigate a more chronic effect of the drugs as opposed to acute exposure. The maximum tolerated concentration (MTC) of drugs was determined and used as the highest concentration. We tested CBZ (MTC: 50 μM), which would typically aggravate seizures, and is thus contraindicated in the treatment of Dravet syndrome [33]. CBZ had either no significant effect or induced a slight increase in the activity of *scn1Lab* morphants (Fig 6A1 and 6A2). Then, CLB (MTC: 150 μM), STP (MTC: 12.5 μM) and TOP (MTC: 200 μM) were tested as they are supposed to ameliorate the seizures [33,34]. It was found that CLB significantly decreased the hyperactivity of *scn1Lab* morphants even after 24 hours of incubation at 4 dpf. Moreover, its effect persisted until 5 dpf (Fig 6B1 and 6B2. Similarly, STP could significantly suppress the hyperactivity of *scn1Lab* morphants after 48-hour incubation at 5 dpf (Fig 6C1 and 6C2). TOP decreased the hyperactivity of *scn1Lab* morphants at 4 and 5 dpf (Fig 6D1 and 6D2). We found that VPA (MTC: 100 μM) could also significantly decrease the total movement of *scn1Lab* morphants at 4 and 5 dpf (Fig 6E1 and 6E2). Finally, we also tested FA (MTC: 50 μM) and observed significantly decreased locomotor activity of *scn1Lab* morphants at 4 and 5 dpf, (Fig 6F1 and 6F2).

#### Forebrain local field potential recordings

Two of the above-mentioned AEDs were selected for forebrain local field potential recordings, namely fenfluramine (FA), as a promising drug candidate for DS, and sodium valproate (VPA) as a positive control, given the latter has previously been shown to suppress seizures in the *scn1Lab*/*didy* mutant [21].

**Fig 7:**
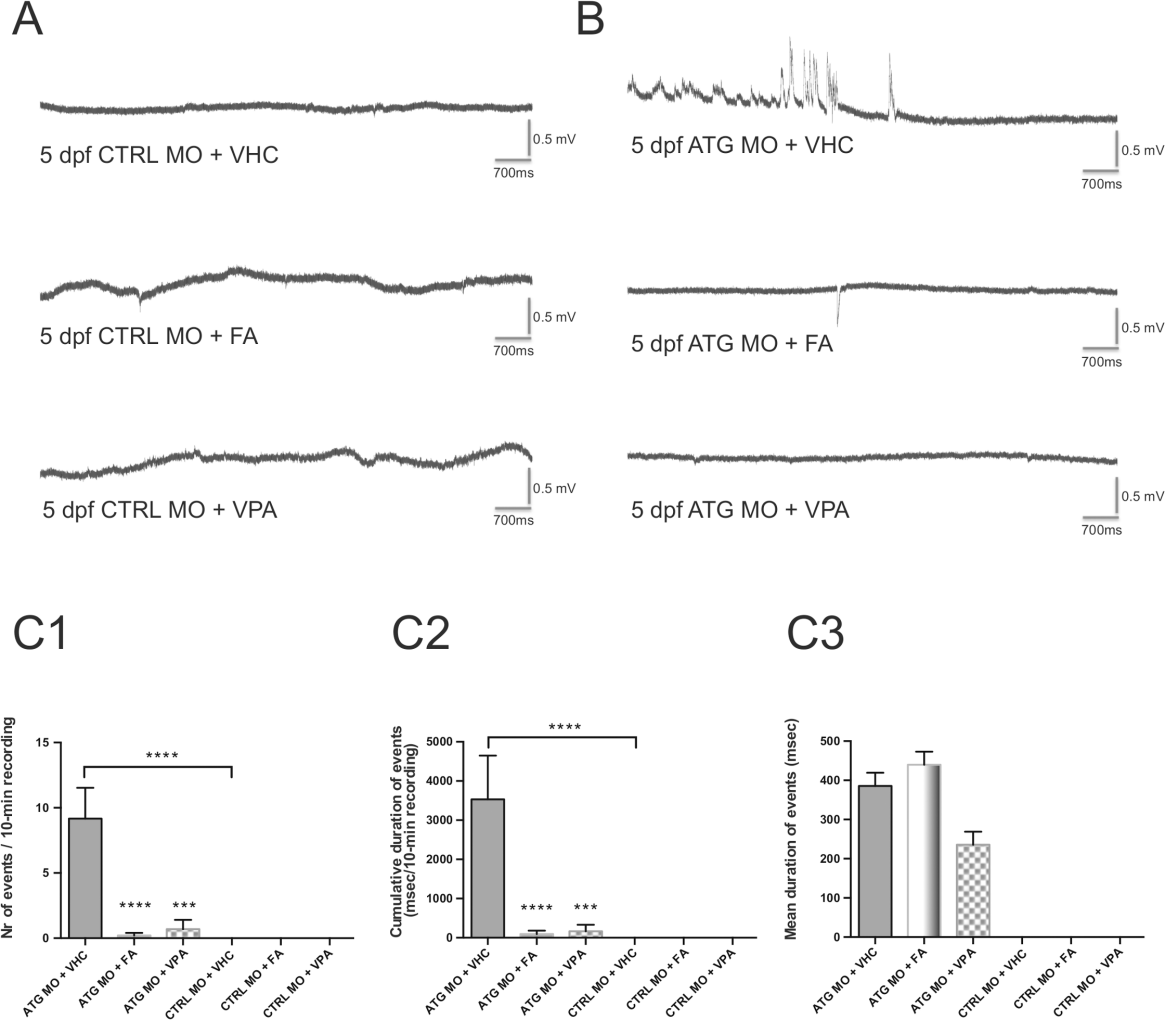
Effect of fenfluramine and sodium valproate on polyspiking discharges recorded from 5 dpf-ATG MO- and CTRL MO-injected larvae. (A) Representative electrographic activities recorded in CTRL MO larvae incubated with vehicle (VHC), 50 μM fenfluramine (FA) or 100 μM sodium valproate (VPA). (B) Representative electrographic activities recorded in ATG MO-injected larvae incubated with the same compounds. (C1) Occurrence of polyspiking discharges recorded in ATG MO-injected larvae after treatment with VHC, 50 μM FA and 100 μM VPA in all larvae. (C2) Cumulative durations of polyspiking discharges recorded in ATG MO-injected larvae after treatment with VHC, 50 μM FA and 100 μM VPA in all larvae. (ATG MO + FA: 0.2±0.2 events and 88±88 msec/10-min recording; ATG MO + VPA: 0.7±0.7 events and 165±165 msec/10-min recording, n = 20 and 10 larvae, respectively) compared to VHC-treated larvae (ATG MO + VHC: 9.16±2.36 events and 3533±1113 msec/10-min recording; n = 12 larvae; (C3) Mean durations of polyspiking discharges recorded in ATG MO-injected larvae after treatment with VHC, 50 μM FA and 100 μM VPA in seizure-positive larvae (ATG MO + VHC: 385.4±34.1 msec; ATG MO + FA: 439.9±33.2 msec; ATG MO + VPA: 235.7±33.2 msec; n = 110, 4 and 7 events analyzed, respectively). Statistical analysis was performed using Student’s unpaired *t*-test or Mann–Whitney test for data that failed the normality test, as appropriate. Error bars on all graphs represent standard error of mean (SEM). *** P≤0.001, **** P≤0.0001.

The effect of fenfluramine on the occurrence of seizures was then examined in 5-dpf *scn1Lab* morphants and control larvae. Application of vehicle was used as a negative control. None of the control larvae displayed any epileptiform activity after incubation with vehicle, 50 μM fenfluramine or 100 μM sodium valproate (Figs 7A and 6C1–6C3). Moreover, application of vehicle had no effect on the frequency (data not shown in Fig 7), cumulative duration and mean duration of events in ATG MO-injected larvae (data not shown in Fig 7).

Application of fenfluramine or sodium valproate induced a dramatic decrease in the occurrence of epileptic events. Indeed, incubation of *scn1Lab* morphants with vehicle induced epileptiform events in 10 out of 12 larvae (Fig 7B) whereas only 1 out of 30 displayed such epileptiform pattern following application of fenfluramine or sodium valproate. The occurrence of polyspiking discharges was significantly decreased after treatment with 50 μM fenfluramine or 100 μM sodium valproate, compared to VHC (Fig 7C1). The cumulative duration of events was significantly decreased as well whereas the mean duration of events was not affected in seizure-positive larvae (Fig 7C2 and 7C3).

#### Hyperthermia-induced abnormalities

**Fig 8:**
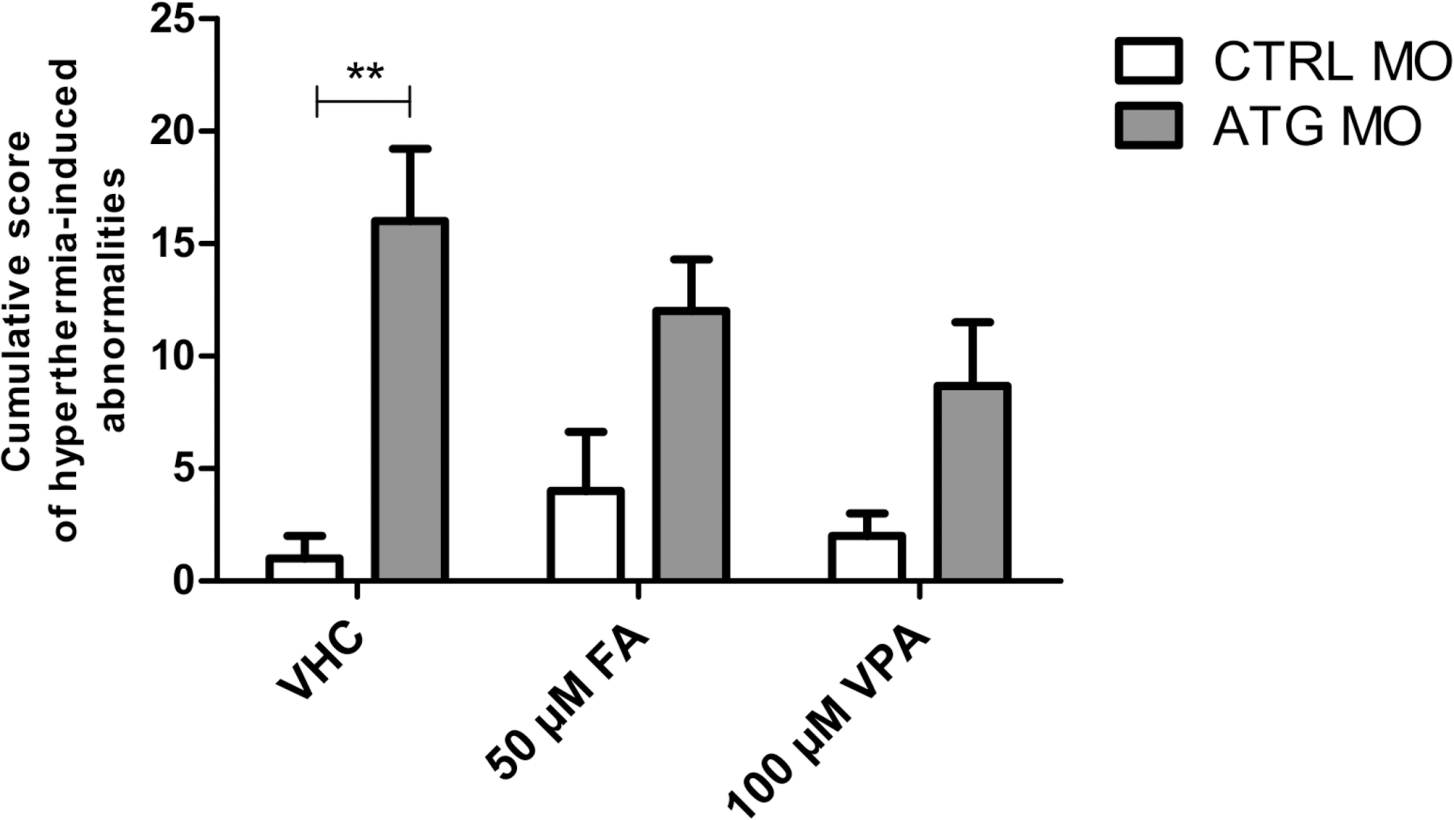
Effect of fenfluramine and sodium valproate on hyperthermia-induced abnormalities on 5 dpf-ATG MO- and CTRL MO-injected larvae. The y-axis depicts the cumulative score to quantify the severity of hyperthermia-induced abnormalities 24 hours after heatshock. Normal behavior was scored as 0, decreased touch response and partial loss of posture as 1, absent touch response and complete loss of posture as 2, and death as 3. The number of larvae was multiplied by the value corresponding to the level of severity. The x-axis corresponds to CTRL MO- and ATG MO-injected larvae at 5 dpf (day of heatshock). 4-dpf *scn1Lab* morphants and control larvae were exposed to the compounds 24 hours prior to heatshock treatment. Data of graphs are pooled together from three independent experiments with 8 larvae per condition. Statistical analysis was performed using two-way ANOVA with Bonferroni post-tests. Error bars on all graphs represent the standard error of mean (SEM). ** P ≤ 0.01.

The potential of fenfluramine and sodium valproate to reduce hyperthermia-induced abnormalities was investigated on 5-dpf *scn1Lab* morphants and control larvae after 24 hours of treatment in comparison to vehicle. Surprisingly, treatment with both fenfluramine and sodium valproate slightly, but non-significantly reduced hyperthermia-induced abnormalities (Fig 8), thereby suggesting that the mechanisms causing these abnormalities are at least in part unrelated to the seizure background that can be rescued by fenfluramine and sodium valproate.

In general, hyperthermia and/or fever are known to induce molecular, structural and functional changes, including increased expression of immune mediators (IL1-β, IL-6, neuropeptide Y), altered ion channel kinetics and axonal conduction velocity, and over-activation of TRPV4-channels. These changes render the normal network hyperexcitable and are associated with febrile seizures [35–37]. It is currently not clear which of these mechanisms are involved in the hyperthermia-induced abnormalities specifically seen in *scn1Lab* morphants under high, non-physiological temperatures. Further analysis will therefore be needed to improve our understanding of the lack of activity of these two drugs under these conditions.

## Conclusion

In this study, we generated a zebrafish model of Dravet syndrome through the antisense-mediated morpholino knockdown of the zebrafish *scn1Lab* gene. We show that *scn1Lab* morphants display hyperactivity, abnormal myoclonic-like behavior and epileptiform brain activity. Furthermore, it was also shown that these larvae are remarkably sensitive to hyperthermia. Pharmacological evaluation revealed that clobazam, stiripentol, topiramate, sodium valproate and fenfluramine significantly decreased the hyperactivity of *scn1Lab* morphants, while carbamazepine, a contra-indicated drug for the treatment of Dravet, had no effect. Moreover, sodium valproate and fenfluramine significantly reduced epileptiform discharges in *scn1Lab* morphants. Furthermore, the Dravet morphant phenotype is remarkably comparable to that of *scn1Lab* mutants with no additional dysmorphologies, and normal control MO knockdown larvae, suggesting that the effects of the ATG MO are highly specific. Altogether, we are able to phenocopy the *scn1Lab* mutant, which is generally accepted as the major criterion for demonstrating knockdown specificity [38].

The *scn1Lab* morphants described here have certain advantages as a zebrafish model of Dravet syndrome in comparison to *scn1Lab* mutants. One advantage is that there is no need to breed mutants over several generations to obtain homozygosity, and no need to genotype all larvae in order to identify homozygotes. In addition, *scn1Lab* morphants show high penetrance (>80%), enabling experiments to be performed on nearly all knockdown larvae at early developmental stages (i.e. prior to the hyperpigmentation phenotype observed from 3 dpf onwards). This contrasts with the 4-fold lower number of embryos with a Dravet-like phenotype for *scn1Lab* mutants, as heterozygous crosses produce only 25% homozygous recessive mutants per mating. Moreover, it is possible to introduce morpholinos into various transgenic reporter lines to easily and rapidly investigate the behavioral, cellular and molecular aspects of Dravet syndrome further.

To our knowledge, this is the first study demonstrating effective seizure inhibition of fenfluramine in an animal model of Dravet syndrome. The high efficacy of fenfluramine in reducing convulsions and epileptiform activity in *scn1Lab* knockdown zebrafish larvae demonstrates their utility as an animal model of Dravet syndrome. This novel zebrafish Dravet model will be useful in identifying and elucidating novel mechanisms of action of potentially therapeutic small molecules, for drug repurposing screens, and for disentangling the mechanisms underlying cardiotoxicity and the anticonvulsant activity of fenfluramine. Thereby we underscore the validity of our model as a rapid first-pass screening tool in assessing the anticonvulsant activity of novel analogs with improved activity and significantly milder or no side effects that can potentially be used therapeutically in Dravet syndrome children.

## Supporting Information

S1 Video A representative 5 dpf *scn1Lab* morphant. (AVI)

S2 Video A representative 6 dpf *scn1Lab* morphant. (AVI)

S3 Video A representative 5 dpf control larva with non-inflated swim bladder (NISB CTRL). (AVI)

S4 Video A representative 6 dpf control larva with non-inflated swim bladder (NISB CTRL). (AVI)

S5 Video A representative 5 dpf control larva. (AVI)

S6 Video A representative 6 dpf control larva. (AVI)
